# A fast, easy and reliable method for hamstrings graft size prediction in anterior cruciate ligament reconstruction

**DOI:** 10.1007/s00167-023-07510-z

**Published:** 2023-07-19

**Authors:** C. Minoli, M. Travi, C. Monti, P. Ferrua, Marco Puce, S. Radaelli, A. Menon, A. L. Tassi, P. S. Randelli

**Affiliations:** 1 U.O.C. Week Surgery, ASST Centro Specialistico Ortopedico Traumatologico Gaetano Pini-CTO, Piazza Cardinal Ferrari 1, 20122 Milan, Italy; 2 Department of Reconstructive Surgery of Osteo-Articular Infections, IRCCS Istituto Ortopedico Galeazzi, 20100 Milan, Italy; 3 Scuola di Specializzazione in Ortopedia e Traumatologia, Università degli Studi di Milano, Via Festa del Perdono 7, 20122 Milan, Italy; 4 MD PhD, Post-Graduation School in Radiodiagnostics Università degli Studi di Milano, Milan, Italy; 5 U.O.C. 1 Clinica ortopedica, ASST Centro Specialistico Ortopedico Traumatologico Gaetano Pini-CTO, Piazza Cardinal Ferrari 1, 20122 Milan, Italy; 6 Laboratory of Applied Biomechanics, Department of Biomedical Sciences for Health, Università degli Studi di Milano, Via Mangiagalli 31, 20133 Milan, Italy; 7 Scuola di Specializzazione in Statistica Sanitaria e Biometria, Dipartimento di Scienze Cliniche e di Comunità, Università degli Studi di Milano, Milan, Italy; 8 Research Center for Adult and Pediatric Rheumatic Diseases (RECAP-RD), Department of Biomedical Sciences for Health, Università degli Studi di Milano, Via Mangiagalli 31, 20133 Milan, Italy

**Keywords:** Knee, ACL reconstruction, Graft, Size, Arthroscopy

## Abstract

**Purpose:**

The aim of this study is to describe and validate a simple and reliable method to pre-operatively predict the size of the ACL graft in the double strand technique with autologous semitendinosus–gracilis tendons on the same MRI used for ACL rupture diagnosis.

**Methods:**

The study included 92 patients, with a median age of 31 years (IQR 26–41 years), 73/92 (79%) of whom were males. All patients that underwent an ACL reconstruction with doubled ST + GT between 2017 and 2022 were counted in the study.

**Results:**

Overall, the median predicted graft diameter from MR imaging was similar to the actual graft diameter with no significant differences (n.s.). Regarding the comparison between predicted and actual graft size, concordance was 78/92 (85%, 95% CI 76–91%), with *κ* = 0.797 which corresponds to a level of agreement defined as “Strong”. Tendon sizes calculated on pre-operative MRI were evaluated both with intra-observer and inter-observer reliability demonstrating a statistically reproducible method. The predicted graft was then compared to the reported one with a statistically significant reliability found.

**Conclusion:**

This study can help the surgeons to perform a fast pre-operative planning of an ACL reconstruction for graft selection. If the planned graft with ST and GT is smaller than 8 mm, the clinician can decide to switch to a different type of graft or plan a different graft preparing technique and, therefore, reduce the risk of post-operative ligament re-rupture. The method proposed is reliable and reproducible. The major strength of the planning technique proposed is that it relies on data that are already available for the clinician before surgery, without the need of further analysis.

**Level of evidence:**

IV.

## Introduction

ACL reconstruction (ACLR) is, therefore, one of the most common procedures performed in orthopaedic surgery [26].

Different grafts are used for the ACLR, mainly autologous tissues such as Hamstrings tendons (semitendinosus and/or gracilis), bone–patellar tendon–bone or quadriceps tendon [8, 16, 22].

The standard procedure for preparing a hamstrings tendon (HT) graft require to flip both tendons in half obtaining a four-bundle graft. This technique, although easy and fast, has one major downside: the size of the graft cannot be decided by the surgeon because ST and GT are harvested as one single structure [5, 34].

The graft diameter is patient dependent and may vary due to the individual biometric characteristics of the patient (height, weight, bone structure, sex, ethnicity, etc.) [6, 18, 23].

The overall ACLR re-rupture rate in literature ranges from 3% up to 25% and is around 8% at 2 years post-op [3, 14, 30, 33, 34].

One of the risk factors for ACL re-rupture is an insufficient graft diameter, leading to a weaker neo-ligament. Different studies in literature have shown that an increased risk of re-rupture is statistically related to an ACL reconstructed with a graft with a diameter of 7 mm or less [7, 15, 29, 32, 33].

The necessity to have a graft large enough led to the development of an alternative technique for graft preparation or pre-operative planning.

Knowing pre-operatively the size of the planned graft could help the surgeon to different graft choices or different graft preparation techniques to maximise the outcome of the reconstruction.

Most known procedures at the present time for pre-operative planning require techniques that, even if accurate, are impractical and difficult to apply in a clinical field [2, 20, 25].

The aim of the study is to propose and validate a simple and reliable method to pre-operatively predict the size of the ACL graft in the double strand technique with autologous ST–GT tendons.

The technique proposed is meant to be performed on the same MRI used for ACL rupture diagnosis, therefore making it available in most clinical settings.

The hypothesis of this study is that the propose method for ACL graft diameter prediction is reliable and reproducible.

## Materials and methods

The Institutional Review Board approved the study protocol (authorization Fondazione IRCCS Ca’ Granda Ospedale Maggiore Policlinico—Milano Area 2, Lombardia, Milan (n°848_2021, Milan, 14.09.2021).

The study included 92 patients, with a median age of 31 years (IQR 26–41 years), most of the patients was males (73/92 or 79%).

All patients that underwent an ACL reconstruction with doubled ST + GT between 2017 and 2022 were included in the study.

Only patients who had known final diameter of the graft were selected. For all those patients, the pre-operative MRI was researched. Amongst those patients, only those that had an MRI performed during the investigation centre were considered eligible for the study. All MRI analysed were performed with the same 1.5 T machine and the same imaging reconstruction protocol. The measurements were performed by two independent researchers at time 0 and then repeated after 1 month to assess the inter- and intra-observer reliability of the proposed pre-operative planning method. All measures were performed using the same programme (Impax Client).

The exclusion criteria for the study were as follows: neoplastic disease around the knee; radiological evidence of surgical procedures in the affected limb; any synthetic material in the affected limb; age less than 18 years old; open physis and past fractures around the knee.

The collected data for the study were: initials; age at the moment of the MRI; gender; affected side; date of the surgical procedure; graft diameter as reported on the surgical report; major diameter of ST; minor diameter of ST; major diameter of GT; minor diameter of GT.

From the collected data, for each patient, the mean diameters of both the ST and GT and pre-operative graft diameter predicted were calculated.

The pre-operative predicted graft size and the intraoperative graft size reported were then confronted.

The intra- and inter-observer reliability was then calculated between the major and minor diameter of the tendon measured by the two researchers at time 0 and 1 month after.

The predicted graft size was then compared with the researchers to assess if, even if there were differences in the measurements of the single tendons diameters, the overall result of the predicted graft size was affected or not.

### Measuring method

**Fig. 1:**
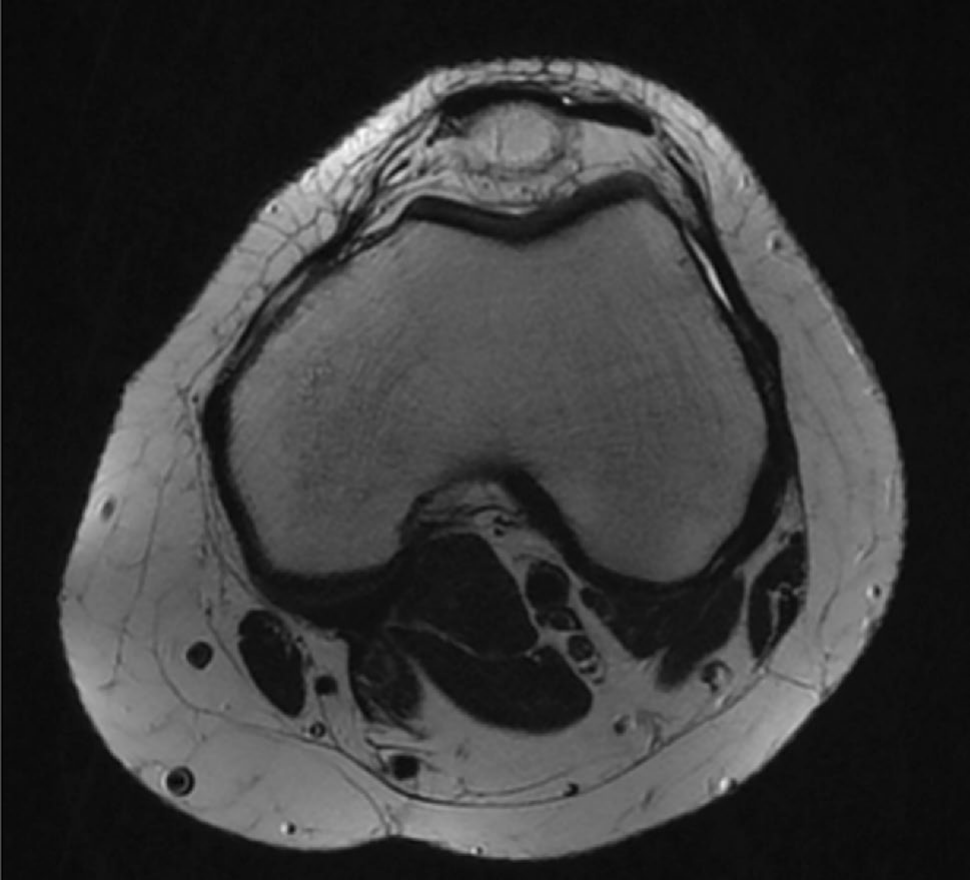
Transverse T2 fat sat cut with the biggest area of the epicondyles present

The measures were performed in the pre-operative MRI. Transverse cuts in T2 fat sat, PD or STIR protocols were evaluated. One slice for each patient was used for the measurement. The image slice where the epicondyles were more represented was selected. After this first selection, the slices above and below were also evaluated to select the one where the ST and GT tendons were more round shaped (Fig. 1).

**Fig. 2:**
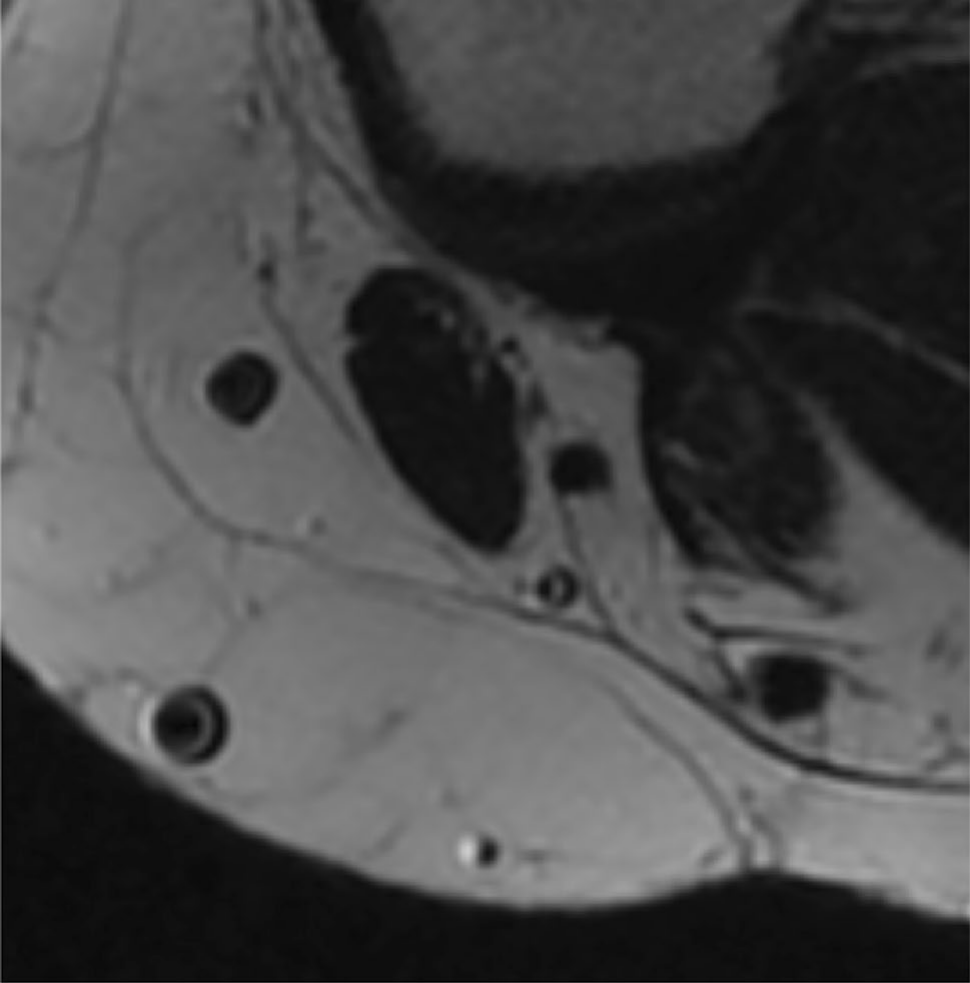
Focus on the transverse section on semitendinosus and gracilis on transverse T2 fat sat cut

**Fig. 3:**
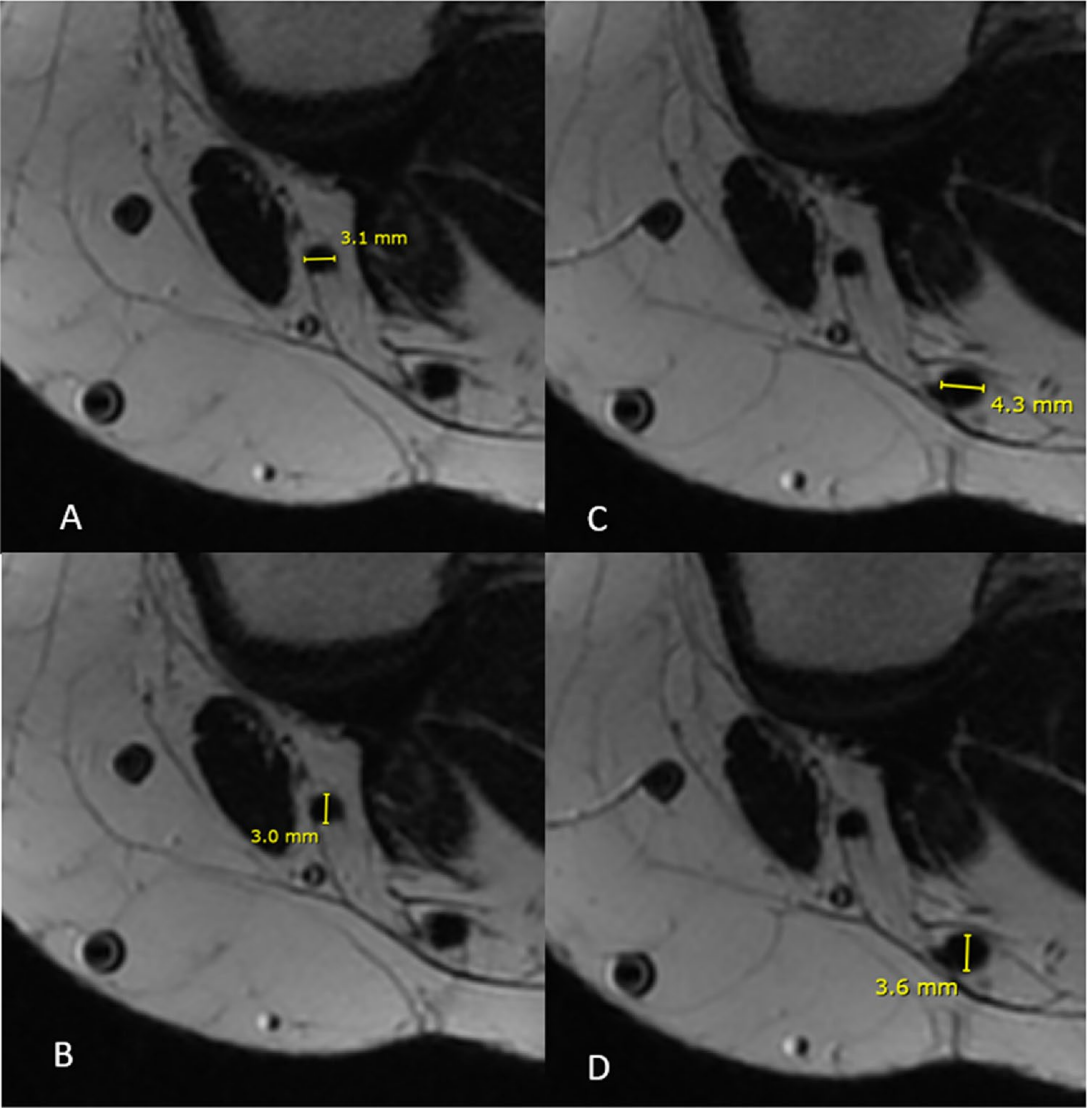
**A** Major diameter of gracilis tendon; **B** minor diameter of gracilis tendon; **C** major diameter of semitendinosus tendon; **D** minor diameter of semitendinosus tendon

Once selected the slice, ST and GT major (*D*) and minor (*d*) diameters were measured in mm (one decimal approximation). The measurement of tendons was carried out with one decimal place taken into account due to limitations in the measurement programme. However, the mathematical formula used later was adjusted to include two decimal places, enabling a more precise calculation of the mean tendon diameter (*D* + *d*/2) and consequently leading to a more accurate prediction of graft size (Figs. 2, 3).

For each tendon, the mean diameter was calculated ((*D* + *d*)/2). Then the mean diameter of ST and GT was added, to obtain the predicted MRI graft diameter. The final measure obtained was then rounded up by excess to the next full millimetre. The rounding was performed to the higher full diameter because, intraoperatively, the graft can fit through a bigger tunnel and not in a smaller one.

$$\mathrm{PGD}=\frac{D\mathrm{St}+d\mathrm{St}}{2}+\frac{D\mathrm{Gr}+d\mathrm{Gr}}{2}$$

$$\mathrm{PGD}\to \mathrm{excessive \, rounding}$$

PGD: predicted graft size; D: major diameter; d: minor diameter; St: semitendinosus; Gr: gracilis.

### Statistical analysis

Data were reported as median and interquartile range (IQR) when quantitative, and as counts and percentages when qualitative. Differences between quantitative data distributions were evaluated with the Wilcoxon test. For both ST, GT and overall graft size, intra- and inter-reader reproducibility were appraised via Bland–Altman analyses and reported as bias and coefficient of repeatability (CoR). The agreement between predicted and actual graft size was assessed with raw concordance, 95% confidence intervals (CI) and linearly weighted Cohen’s *κ*, and interpreted accordingly [21].

Statistical analyses were conducted using Python 3.7.6, and *p* values < 0.05 were chosen as a threshold for statistical significance.

No sample size was calculated for the study, but rather all consecutive patients referred for pre-operative magnetic resonance imaging (MRI) at the research centre before anterior cruciate ligament reconstruction between 2017 and 2022 was retrospectively retrieved.

## Results

### Study population

Considering the entire patient group, in 48/92 (52%) patients, the left ACL was scheduled for reconstruction, whereas in 44/92 (48%) of patients, the right ACL was considered.

Overall, the median predicted graft diameter from MR imaging was 7.5 mm (IQR 6.89–7.90 mm), whilst the actual graft diameter was 8.0 mm (IQR 7.00–8.00 mm), with no significant differences (n.s.).

### Graft measurement reproducibility

**Fig. 4:**
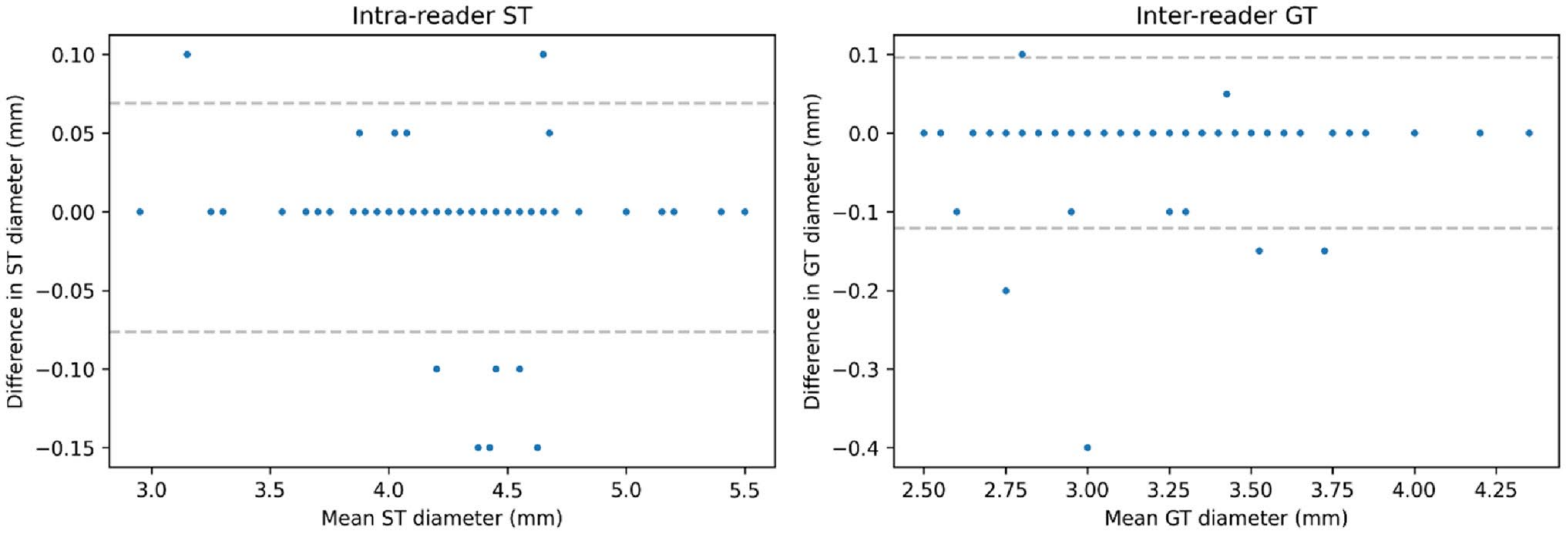
Bland–Altman plots for intra-reader reproducibility for the semitendinosus tendon (ST) and gracilis tendon (GT)

Concerning intra-reader reproducibility, ST diameter displayed a bias of 0.00 mm and a CoR of 0.07 mm, whilst GT diameter displayed a bias of 0.00 mm and a CoR of 0.06 mm. Bland–Altman plots for intra-reader reproducibility are reported in Fig. 4.

**Fig. 5:**
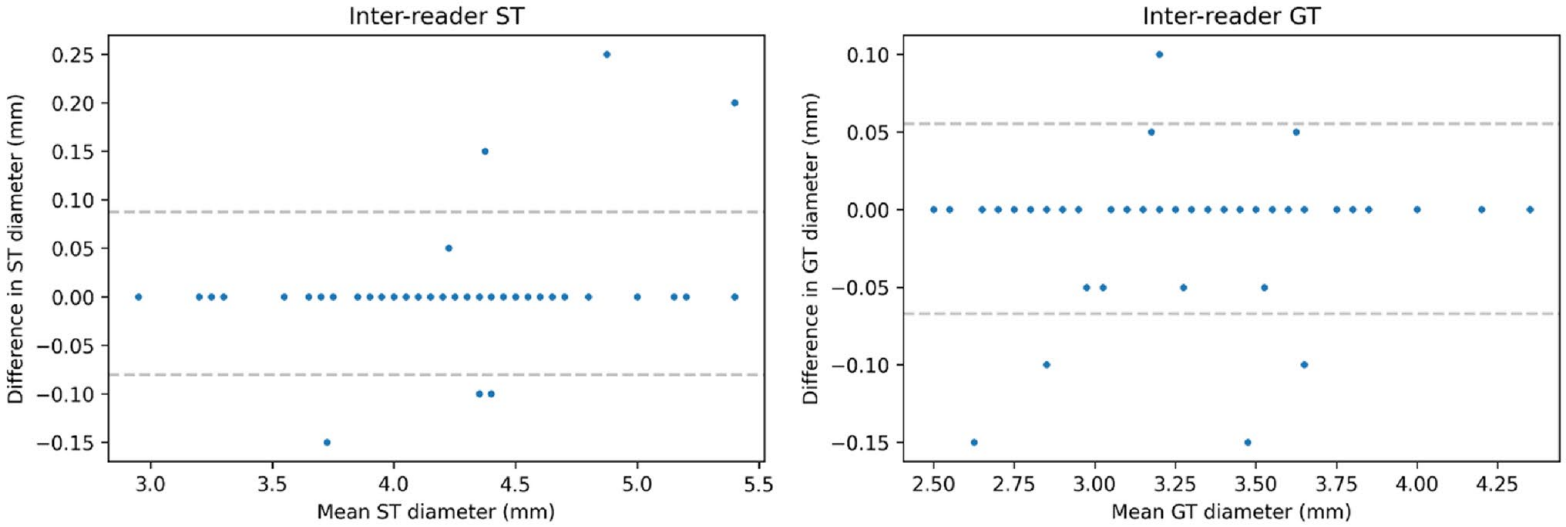
Bland–Altman plots for inter-reader reproducibility for the semitendinosus tendon (ST) and gracilis tendon (GT)

Concerning inter-reader reproducibility, ST diameter displayed a bias of 0.00 mm and a CoR of 0.09 mm, whilst GT diameter displayed a bias of − 0.01 mm and a CoR of 0.11 mm. Bland–Altman plots for intra-reader reproducibility are reported in Fig. 5.

**Fig. 6:**
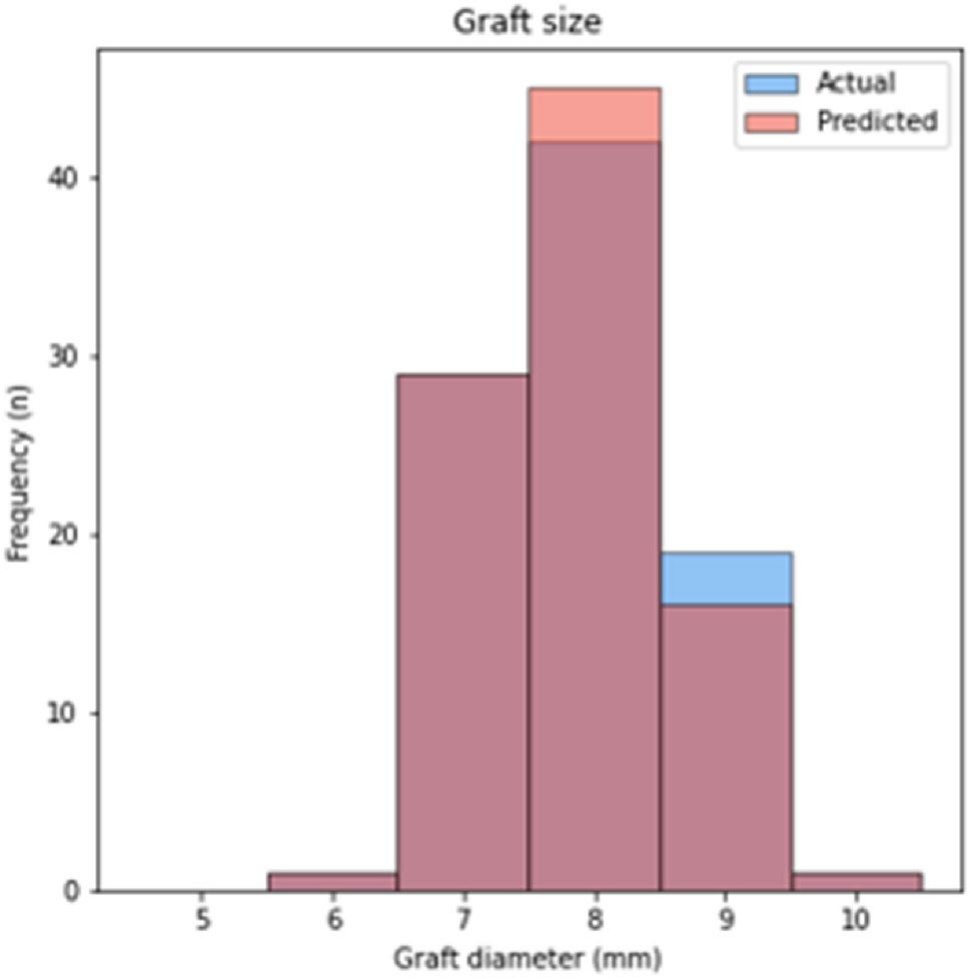
Frequencies of actual and predicted total graft size

Regarding the comparison between predicted and actual graft size, concordance was 78/92 (85%, 95% CI 76–91%), with *κ* = 0.797 which corresponds to a level of agreement defined as “Strong”. In 8/92 cases (9%, 95% CI 4–16%), the predicted graft size was smaller than the actual graft size, whilst in the remaining 6/92 cases (6%, 95% CI 2–14%), the predicted graft size was greater than the actual graft size. The distributions of predicted and actual graft sizes are reported in Fig. 6.

Concerning cases with a predicted graft size smaller than the actual graft size, the difference was 1 mm in all instances, with 5/8 (63%) cases with a predicted diameter (after rounding) of 7.00 mm versus an actual diameter of 8.00 mm, and 3/8 (37%) cases with a predicted diameter (after rounding) of 8.00 mm versus an actual diameter of 9.00 mm. In all these cases, patients were males with a median age of 27 years (IQR 24–30 years), whilst the ACL lesion was on the left side in 5/8 (63%) cases and on the right side in 3/8 (37%) cases, with no significant differences (n.s.).

## Discussion

The main finding of this study is that the proposed method for pre-operative evaluation of expected graft diameter in ACL reconstruction with ST–GT is reliable and reproducible.

In ACL reconstruction surgery, the importance of surgical planning is supported by the evidence that up to 8.2% of ACLR end in graft failure [7, 9, 11, 19, 22, 29, 34].

Multiple causes could lead to an ACL re-rupture including non-compliance to the rehabilitation protocol, traumas, patients’ selection, or surgical error.

One of the risk factors reported in literature for neo-ACL rupture is the reconstruction with a graft of insufficient diameter. Eight millimetre is reported as the threshold under which the risk of ACL re-rupture is statistically higher [12, 17, 29].

Amongst the graft that the surgeon can decide to use, ST and GT have one major disadvantage over the others: the size of the graft is patient dependent and cannot be selected by the surgeon.

Different studies are present in the literature showing the possibility of successful graft size prediction using analysis of MRI images of the knee both in children [27, 31] and adults [4, 13, 28].

Nearly every one of them succeeded, supporting the theory that a pre-operative planning is possible.

The previously reported techniques, even if accurate, are complex to perform, requesting elaborated mathematical calculations, and therefore fail to present a clinically feasible planning method.

Furthermore, most of the proposed systems have not been validated by the inter- and intra-observer reliability.

Other studies tried different imaging techniques or planning performed on anthropometric parameters with poorer results [1, 2, 10, 24, 31].

These studies require a pre-operative planning based on exams that are not necessary for the diagnosis nor the treatment of a patient with an ACL rupture, therefore representing a waste of time and resources either for the patient or the national health system.

The authors’ idea was that all patients with a diagnosis of ACL rupture must have performed at least one MRI; therefore, this was the imaging method that the authors choose for the planning.

The evaluation method proposed has been found to be reliable and reproducible.

This method, although reliable, presents two main limitations.

The first limitation is that the study has been performed with strict inclusion criteria that allowed only the selection of patients that performed the knee MRI at the research centre, with the same machine, the same image reconstruction protocol and the same measuring programme (Impax Client). More studies performed in different centres with different programmes should be performed to confirm the effectiveness of the proposed pre-operative planning method.

The authors selected for this study a measuring programme that allowed for millimetre decimals (e.g. 0.1 mm). The measures of the tendons calculated are small, and a programme that automatically rounds one decimal, instead all mathematical calculations rounded to two decimals.

The second limitation is that the study was retrospectively designed; therefore, it was not possible to directly evaluate the graft size reported in the surgical report. There is one patient in the study database that has a 2-mm difference between the predicted size (9 mm) and the reported size (7 mm).

The main strength of this study is that the proposed method is highly reproducible, as confirmed by the statistically significant inter- and intra-observer reliability.

The results of this study could help the clinicians to perform a fast pre-operative planning of an ACL reconstruction regarding graft selection. If the planned graft with ST and GT is smaller than 8 mm, the clinician could decide to switch to a different type of graft or plan a different graft preparing technique and, therefore, reduce the risk of post-operative ligament re-rupture.

## Conclusion

The method proposed is reliable and reproducible. The major strength of the planning technique proposed is that it relies on data that are already available for the clinician before surgery, without the need for further analysis or complex mathematical formulas. This method will help the clinician to select the graft or the graft preparing technique before surgery.
